# 

**Published:** 1896-11

**Authors:** 


### TO THE MEMBERS OF THE STATE MEDICAL ASSOCIATION.

The next meeting of our Association will be held in Macon, a delightful city and a central point. The "good fellows" there are sure to make the meeting pleasant socially, and the officers of the Association hope to have a good lot of papers to make it a successful one. We hope that members will begin to think over the subjects for papers, write the papers, and make all their arrangements with a view to being there.

THE ninth annual meeting of the Southern Surgical and Gynecological Association, will be held in Nashville, Tenn., Tuesday, Wedneday, and Thursday, November 10th, 11th, and 12th, 1896. The Nicholson House has been selected as headquarters for the Association. The rates will range from $2.00 to $4.00 per day. Arrangements have been made with the Southern Passenger Association for reduced rates on the certificate plan. These certificates, properly signed, will entitle those who attend to a return ticket at the rate of one-third of one full fare. Those who contemplate attending the Pan-American Medical Congress, to be held in the City of Mexico, November 16-19, will have time to do so after the meeting of the Southern Surgical and Gynecological Association. A rate of one fare for the round trip has been made on account of the Congress, stop-over privileges being allowed holders of tickets therefor. Dr. W. E. B. Davis, Birmingham, Ala., is secretary.

PUBLISHED BY THE FRANKLIN PRINTING AND PUBLISHING CO., ATLANTA, GA.
Entered at the Post-Office at Atlanta, Ga., as second-class matter.

*We positively cannot send premium to any subscriber who does not pay a year in advance.*

NOTICE.—Our offer of a thermometer to *all* cash-in-advance subscribers is withdrawn. *New* subscribers, paying full year in advance, may still get the thermometer. Book offer still open to all who pay a year in advance. "Stories of a Country Doctor."

